# Replantation versus Prosthetic Fitting in Traumatic Arm Amputations: A Systematic Review

**DOI:** 10.1371/journal.pone.0137729

**Published:** 2015-09-04

**Authors:** Iris A. Otto, Moshe Kon, Arnold H. Schuurman, L. Paul van Minnen

**Affiliations:** 1 Department of Plastic, Reconstructive and Hand Surgery, University Medical Center Utrecht, Utrecht, The Netherlands; 2 The Hand Clinic, Amsterdam, The Netherlands; Harvard Medical School, UNITED STATES

## Abstract

**Background:**

Traumatic arm amputations can be treated with replantation or surgical formalization of the stump with or without subsequent prosthetic fitting. In the literature, many authors suggest the superiority of replantation. This systematic review compared available literature to analyze whether replantation is functionally and psychologically more profitable than formalization and prosthetic fitting in patients with traumatic arm amputation.

**Methods:**

Functional outcome and satisfaction levels were recorded of patients with amputation levels below elbow, through elbow, and above elbow.

**Results:**

Functional outcomes of 301 replantation patients and 172 prosthesis patients were obtained. In the replantation group, good or excellent functional scores were reported in 39% of above elbow, 55% of through elbow, and 50% of below elbow amputation cases. Nearly 100% of patients were satisfied with the replanted limb. In the prosthesis group, full use of the prosthesis was attained in 48% of above elbow and in 89% of below elbow amputation patients. Here, 29% of patients elected not to use the prosthesis for reasons including pain and functional superfluity. In both replantation patients and prosthesis wearers, a below elbow amputation yielded better functional results than higher amputation levels.

**Conclusions:**

Replantation of a traumatically amputated arm leads to good function and higher satisfaction rates than a prosthesis, regardless of the objective functional outcome. Sensation and psychological well-being seem the two major advantages of replantation over a prosthesis. The current review of the available literature shows that in carefully selected cases replantation could be the preferred option of treatment.

## Introduction

Traumatic loss of a limb is a devastating event with major functional and psychological consequences. Besides functional challenges, amputation patients face feelings of being disfigured. Patients are often focused on portraying a normal appearance, with the underlying motivation to avoid being rejected by society [1].

A prosthesis can increase a person’s quality of life and body image by improving functional capacity and by cosmetically concealing their deformity. Prostheses have been proven very successful in the rehabilitation after lower limb amputation. It appears, however, that upper limb prostheses are functionally and psychologically less satisfying [1].

Since the first successful reattachment of an amputated arm by Chen *et al* in 1962, replantation has become a viable option in selected cases of upper limb amputation [2]. In the early years, the main concern was for the replant to remain viable. With improved (micro)surgical techniques and instrumentation, replantation has become a technically reliable procedure [3,4]. Presently, the concern has shifted towards obtaining satisfactory functional recovery with an acceptable cosmetic result [5–11].

Still, the question remains whether arm replantation is superior to an amputation stump fitted with an appropriate prosthesis [6,7,9,12]. The issue is controversial as functional outcomes vary between case series and are affected by factors such as the level of amputation, patient age and quality of prostheses.

No systematic reviews on this topic have been published to date. The aim of this literature review is to find a conclusion on whether replantation is functionally and psychologically superior to a prosthesis in patients with traumatic amputation of an arm.

## Methods

### Search

A literature search using the electronic databases MEDLINE, EMBASE and Cochrane was performed, identifying 1034 unique articles. Search terms included “arm”, “forearm”, “upper limb”, “upper extremity”, “trauma”, “traumatic”, “amputation”, “replantation”, “reattachment”, “prosthesis”, “prosthetics” and “function”. The Cochrane Library identified no systematic reviews on this topic. All articles describing the functional and/or psychological outcome in patients with traumatically amputated arms, whether they be replanted or fitted with a prosthesis, were deemed eligible for inclusion.

All patients with a single-level traumatic amputation above, through, or below the elbow were included in this review. Shoulder, wrist, hand and digital amputations were omitted as these levels of injury prove to have markedly different outcomes [13] and should be regarded as separate groups. Prosthesis patients with either body-powered or myoelectric prosthesis were included.

Careful patient selection is considered crucial to a successful outcome in upper limb replantation, and therefore only good candidates for survival of the replant are eligible for the current comparison. Poor candidates include patients with multilevel trauma, comorbidity such as plexus injuries, and mental instability [14], hence such patients were excluded in this review. Replantation cases by arm allotransplantation or cross-arm replantation were excluded. Additional exclusion criteria were non-English language, causes other than trauma, and incomplete amputations. It was ensured that identical patient groups described in separate articles were not included twice. If additional patients were discussed in such an article, said article was included for those patients only.

**Fig 1:**
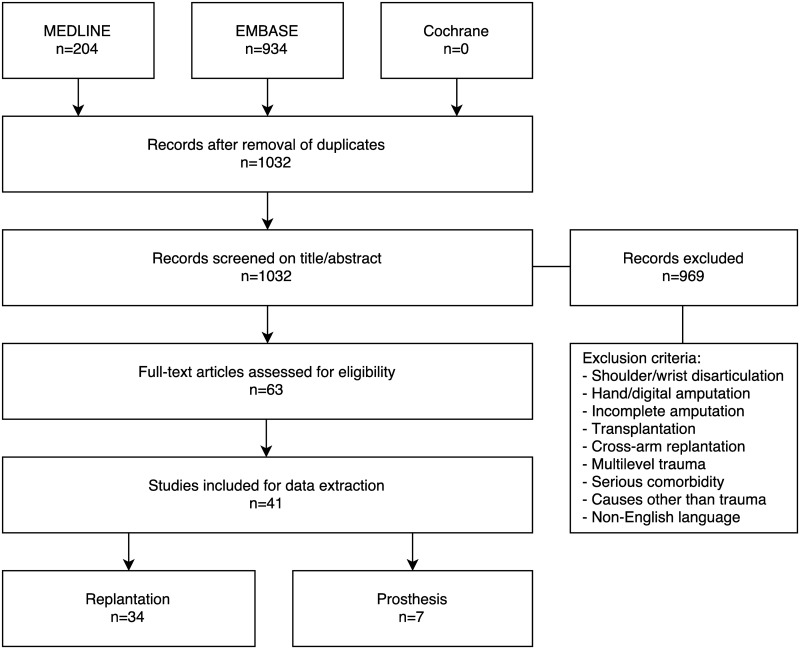
Preferred Reporting Items for Systematic Reviews and Meta-Analyses (PRISMA) flow chart. Identification and selection of articles for inclusion in this systematic review.

After title/abstract screening, 969 articles were excluded for the aforementioned reasons, leading to a total of 63 articles eligible for full-text screening. Of those, 39 were included for data extraction: 34 for replantation and 7 for prosthesis (Fig 1) [2,5–11,13,15–44]. Attempts to contact the author for any missing data on individual results were made in five instances. Unfortunately, most of the original data from last century is no longer available.

Most of the research was done in the form of observational studies, with no controlled trials. This means that mostly evidence level III sources contribute to the results.

### Data extraction

Functional outcomes in replantation cases were measured through Chen’s criteria [45], the Disabilities of the Arm, Shoulder and Hand (DASH) outcome measure [10,46], or comparable measures (UEFT/Carroll test [19,40], Tamai score [28]). Information on motor and sensory function was also noted when no primary outcome measure was available. In patients with prostheses, no specific functional tests were reported. Therefore, usage rate was taken as the parameter for prosthetic functional outcome. Psychological outcome was defined in both groups as the satisfaction level of the patient.

Data on functional and psychological outcomes per individual patient was extracted. Collected data further included patient demographic information, mechanism and level of injury, and length of follow-up period. Mechanism of injury was categorized as sharp, crush, avulsion, or combined. The level of injury was determined as being either *below elbow* (proximal to the wrist), *through elbow*, or *above elbow* (distal to the shoulder). Grouped results were noted and analyzed if individual results were not reported.

### Data analysis

As various functional outcome scores were used in the literature on replantation, these were compared according to their descript criteria [19,28,40,45] and four final scores were set: *excellent*, *good*, *fair* and *poor* (Table 1). Outcomes per patient and per patient group were analyzed. Individual results were converted into a grouped result, and then combined grouped results were assessed. Data was categorized based on amputation level and then scored for functional and psychological outcome.

**Table 1:** Stratification of outcome measures as used in available literature.

Excellent	Good	Fair	Poor
Chen’s level I	Chen’s level II	Chen’s level III	Chen’s level IV
UEFT ≥85	UEFT 75–84	UEFT 51–74	UEFT <51
Tamai 80–100	Tamai 60–79	Tamai 40–59	Tamai 0–39
2PD <19mm	2PD 20-24mm	2PD 25-29mm	2PD >30mm
Protective sensation	Protective sensation	Some sensation	No sensation
Powerful pinch/grip	Useful pinch/grip	Weak pinch/grip	Absent pinch/grip
Full range of motion	Useful flexion	Used as assisting hand	Not useful

Available literature on prosthesis patients was limited. Functional outcome was predominantly expressed in usage rate. Hence, functional outcome this was scored according to use: *full use*, *limited use*, and *no use*.

## Results

### Study characteristics

Individual results were extracted for 122 patients from 26 articles in the replantation group, and 5 patients from 3 articles in the prosthesis group. Grouped results were extracted from 8 replantation articles and 4 prosthesis articles. In the studies included in this review, the number of individual cases varied greatly. In the replantation group, the maximum number of patients in individual descriptions was 16, with a mean of 4.7. The mean in articles describing groups of patients was 31.3, with a range of 2 to 49 patients. In the prosthesis group, the maximum number of individual patients was 3, with a mean of 1.7. In articles describing groups of patients, the mean was 44.5, with a range of 6 to 51 patients.

### Patient demographics

Patients who underwent replantation were significantly younger than patients who had a prosthesis (Table 2). Mean age in the replantation group was 28 years (range 1 to 76 years) in comparison to 42 years (range 2 to 70 years) in the prosthesis group. Mean follow-up also differed greatly, with 64 months for the replantation group and 133 months for the prosthesis group. The great majority of patients was male, respectively 91% and 100% of patients. Most amputations occurred below elbow, and avulsion was the most commonly described mechanism of amputation. Types of prostheses included body-powered (cable operated) and myoelectric prostheses, although not all studies clearly identified which type of prosthesis was applied in the patient.

**Table 2:** Patient demographics. *Please note that data on level and mechanism of amputation are incomplete.

	Replantation	Prosthesis
**Number of patients**	301	172
**Mean age** (years)	28 (1–76)	42 (2–70)
**Sex** (male)	91%	100%
**Mean follow-up** (months)	64	133
Level of amputation*		
above elbow	82	≥74
through elbow	21	≥1
below elbow	198	≥82
Mechanism of amputation*		
sharp	≥17	≥24
crush	≥22	≥22
avulsion	≥51	?
combined	≥24	?

### Functional and psychological outcomes

For replantations, functional results could be extracted from the literature for 301 patients. Scores that were *good* or *excellent* were regarded as a satisfactory functional result. Such results were reported in 39% of *above elbow* amputation cases, 55% of *through elbow* amputation cases, and 50% of *below elbow* amputation cases (Table 3). Poor results were seen in 31% of *above elbow* amputation patients, compared to 18% in both *through elbow* and *below elbow* amputation patients. These results indicate that a large proportion of patients attains satisfactory functional results after replantation, and that replantation gives the best results when amputation level is through or below the elbow.

**Table 3:** Functional and psychological results for replantation. Data sources: 6–11,13,15–19,21–8,30–7,39,40,42,44.

	Function (n = 301)	%	Satisfaction (n = 191)	%
**Above elbow**	Excellent	17	Satisfied	100
	Good	22	Not satisfied	0
	Fair	30		
	Poor	31		
**Through elbow**	Excellent	14	Satisfied	100
	Good	41	Not satisfied	0
	Fair	27		
	Poor	18		
**Below elbow**	Excellent	20	Satisfied	99
	Good	30	Not satisfied	1
	Fair	32		
	Poor	18		

Satisfaction rates were described in 191 out of 301 replantation patients. With the exception of a single *below elbow* amputation patient, all patients were either satisfied or highly satisfied with the replanted arm, regardless of the defined functional outcome.

In replantation patients aged 20 years or younger, satisfactory functional result were obtained in over 60%. *Excellent* scores became less frequent as patients progressed in age, although still nearly 50% of patients aged 50 or higher obtained a *good* score. The poorest results were achieved in age group 31–40 years, where only 22% scored satisfactory functional results.

In prosthesis patients, *full use* was attained in 48% of *above elbow* amputation patients and in 89% of *below elbow* amputation patients (Table 4). Rejection rates differed greatly with 51% in *above elbow* amputation patients and only 8% in *below elbow* amputation patients. When taking the arm as a whole, 29% of patients rejected or stopped using the prosthesis for various reasons, including discomfort, pain, and functional superfluity [43,47,48].

**Table 4:** Usage rates of prostheses. Data sources: 20,26,30,37,38,41,43.

	Above elbow (n = 82)	%	Below elbow (n = 74)	%	Upper limb (n = 172)	%
**Full use**	39	48	66	89	114	66
**Limited use**	1	1	2	3	8	5
**No use**	42	51	6	8	50	29

## Discussion

The literature describes that most traumatic amputation patients strongly desire to have their arm replanted even though there might be poor function in the long term [5,8–10,15,22,23,42,49]. Although a prosthesis is a good option for upper limb amputation patients, it is often merely seen as an instrument helping the intact opposite arm instead of being able to at least partially replace the function of the amputated arm [37,50,51]. In the era of body-powered prostheses, where rejection rates up to 68% were found, it was speculated that the provision of myoelectric prostheses could increase the number of users to over 90% [41]. However, despite significant progress made in the field of prosthetics, many sources claim that replantation is still superior to a prosthesis [4,5,8,10,13,16,19,23,37].

This review leans to support the assertion of functional superiority of replantation over a prosthesis. Taking into account the very high satisfaction rates in replantation cases, psychological superiority of replantation is suggested as well. However, in patients treated with replantation of the amputated arm, differences in functional outcome were observed in varying levels of amputation. For through or below the elbow replantations, more than fifty percent attained good or excellent scores and only one patient had a poor functional result. In contrast, one-third of amputations above the elbow scored poorly. In general, about half of the replanted arms gained satisfactory functional return when scored objectively. Yet, the great majority of patients expressed their deep satisfaction with the replanted limb and its functional capacity.

Although there is only very limited data available on the functional capacity of prosthesis users, their satisfaction with the prosthesis and its functionality can be estimated by usage rate. Of the analyzed group of prosthesis users, 30% stopped using the prosthesis, which could be interpreted as dissatisfaction with functionality.

These results would favor replantation as the most rewarding intervention in terms of function and patient satisfaction in carefully selected cases of traumatic amputations of the upper limb where replantation is a viable option. Here, selection implies the careful consideration of only patients with single-level trauma, an intact amputated limb, and no serious comorbidity such as plexus injuries. Even though not all patients regained excellent or good function of their replanted arm, as determined by sensory and motor function, practically all expressed satisfaction with the replanted limb and many would even choose the same intervention if having to make the decision again [5,8–10,15,22,23,42,49].

Formalization of the amputation and subsequent prosthetic rehabilitation has the potential to regain satisfactory functionality, restore body image, and return to previous employment [38,52]. It has been used for decades as the standard intervention following traumatic amputation. However, in concordance with the results of this review, it appears that there is an unsatisfactory usage rate for a substantial number of prosthesis users despite the potential benefits [52]. Decreased prosthesis use is reported to be associated with discomfort and increased residual limb pain [20,43,48,52–54]. Additional reasons for low usage rates vary from poor training and late fitting to weight, cost, and maintenance [15,43,47]. A prosthetic limb is often seen as burdensome and non-intuitive [55], with some even stating that a prosthesis could never replace the normal arm when it comes to function [50]. Indeed, there is a vast difference in the functional capability of a prosthesis and a normal arm. Prehension and sensation are arguably the two most important functions of the human hand and arm. Unfortunately, these are currently still almost impossible to replace with modern technology. Despite all the technical advances, prosthetics can only reproduce a fraction of the sensory feedback and range of motion of a normal arm [55]. In contrast, even a replanted arm with poor motor function could have a little sensitive conduction, thereby greatly enhancing overall functionality. Besides the obvious benefit of sensation, replantation also has psychological advantages; various studies have shown that patients with replanted arms have a better sense of self and feel less disfigured than they would without their replanted limb [1,56]. These potential benefits of arm replantation are supported by the fact that current analysis of available data shows that all patients are very satisfied with their replanted limb, regardless of their functional outcome.

This literature review offers an extensive analysis of the available literature on both replantation and prosthetic fitting following traumatic arm amputation. It is the first systematic review to date comparing both functional and psychological outcome in arm amputation patients. Clear inclusion and exclusion criteria have been set in order to create a homogenous study group with respect to level of amputation and type of intervention. As included studies used different functional measures, the various described outcome measures have been stratified to make study data comparable. The aim of the present paper was to assist in the discussion and decision making process in acute trauma situations when confronted with an upper arm amputation. Therefore, we did not include hand transplantation in the present study. Transplantations are planned electively after careful selection and workup after the patient has recovered from initial revision of the amputation. In addition, below the wrist and digital amputations demonstrate markedly different functional outcomes, and these levels of amputation are therefore unsuitable for comparison in the current study.

Since traumatic arm amputations are relatively rare, most of the current literature is based on small study groups or single cases. This review provides the most significant study group to date by pooling all individual replantation and prosthesis patients into a single group and stratifying the various outcome measures. Even so, there is a significant difference between the size of the study groups, with 301 replantation patients and 172 patients with a prosthesis. Additionally, no proper outcome measures were used in the literature to evaluate functional outcome in prosthesis patients. In order to be able to draw more reliable conclusions on whether replantation is favorable over prosthetic fitting, more objective research should be conducted on the latter primarily.

Creating one large study group with patients from various countries and decennia leads to heterogeneity of the used surgical techniques [1] and quality of prostheses. Techniques and materials have most certainly improved over the years, as well as there have been advances in perioperative care, preservation of the amputated part and rehabilitation. These factors will undoubtedly have influenced the results of replantation of severed parts over the past decades. The same can be stated for prostheses, with the shift of body-powered towards myoelectric prostheses, and the alterations made in material, time of fitting, and rehabilitation. The studies available did not always clearly state what type of prosthesis was used; hence, remarks on outcomes with regards to the type of prosthesis could not be made here.

The heterogeneity in the use of functional outcome measures obviates the need for the use of a standard outcome measure in patients with traumatic arm amputations. Although the Chen system is the most widely used to evaluate functional outcome, a wide array of different outcome measures are used in the literature with great differences in what functions were tested. This review shows that psychological outcome is a major factor in the success of replantation, and should therefore be incorporated in an evaluation system. The Disabilities of Arm, Shoulder and Hand score has already been suggested as a suitable standard [10].

The available literature analyzed in this review includes many case reports and case series, meaning that inevitably, the conclusions drawn in this article source mainly from level III evidence. Regardless, it was deemed essential to review the available literature to date to contribute to conclusions on the best therapy for patients with traumatically amputated arms. Statistical analysis was not performed, as this is inaccurate in a heterogeneous pool of level III data sourcing from 50 years of clinical practice. As such, statistical analysis would not contribute to the reliability of the conclusions.

In conclusion, a literature research of the available level III literature indicates that replantation of the traumatically amputated arm leads to good functional outcomes and higher patient satisfaction rates than prosthetic fitting, regardless of the objectively measured functional outcome. In clinical practice this could mean that if technically possible, replantation could be the preferred option of treatment. That being said, careful patient selection and surgical common sense remain the most important parameters in choosing replantation over revision surgery and prosthetic fitting.

### Clinical messages

- Below elbow traumatic amputation has better functional outcome in both replantation and prosthetic fitting.
- Arm replantation benefits from high patient satisfaction rates and few disadvantages that come with prosthetic fitting.
- In selected cases, replantation of the traumatically amputated arm is the preferred option of treatment.

## Supporting Information

S1 PRISMA Checklist (DOC)
